# Humor appreciation of captionless cartoons in obsessive-compulsive disorder

**DOI:** 10.1186/1744-859X-10-31

**Published:** 2011-11-21

**Authors:** Vasilis P Bozikas, Mary H Kosmidis, Maria Giannakou, Aravela Adamopoulou, Xenia Gonda, Kostas Fokas, George Garyfallos

**Affiliations:** 1 First Department of Psychiatry, School of Medicine, Aristotle University of Thessaloniki, Thessaloniki, Greece; 2 Laboratory of Cognitive Neuroscience, School of Psychology, Aristotle University of Thessaloniki, Thessaloniki, Greece; 3 Community Mental Health Center, Northwest District of Thessaloniki, Thessaloniki, Greece; 4 Department of Clinical and Theoretical Mental Health, School of Medicine, Semmelweis University, Budapest, Hungary; 5 Second Department of Psychiatry, School of Medicine, Aristotle University of Thessaloniki, Thessaloniki, Greece

## Abstract

**Background:**

It seems that the core neural regions and cognitive processes implicated in obsessive-compulsive disorder (OCD) pathophysiology may overlap with those involved in humor appreciation. However, to date, there have been no studies that have explored humor appreciation in OCD. The purpose of the present work was to investigate humor appreciation in a group of patients with OCD.

**Methods:**

We examined 25 patients with OCD and 25 healthy controls, matched by age, education, and gender. We administered Penn's Humor Appreciation Test (PHAT), a computerized test comprising captionless cartoons by Mordillo. Each set of stimuli consisted of two almost identical drawings, one of which was funny due to the alteration of a detail in the cartoon, whereas the other was not funny. Severity of psychopathology was evaluated with the Yale-Brown Obsessive Compulsive Scale (Y-BOCS).

**Results:**

No significant effect for group, gender or group × gender interaction was found on the PHAT scores. In OCD patients, humor appreciation was not significantly associated with age of onset, duration of illness, and obsessions, but correlated significantly with compulsions.

**Conclusions:**

Humor appreciation, based on captionless cartoons in OCD, does not seem to be deficient compared to healthy subjects but may be related to illness characteristics.

## Introduction

Data from structural and functional imaging studies in obsessive-compulsive disorder (OCD) have suggested abnormalities in the orbitofrontal cortex, anterior cingulate, caudate, and thalamus, structures linked by well described neuroanatomic circuits [1,2]. Findings of previous studies addressing neuropsychological functioning in patients with OCD, however, have not presented a clear and specific neuropsychological profile for these patients. There is some evidence for visuospatial and verbal memory dysfunction in OCD, owing to an underlying executive deficit in the ability to organize necessary strategies to encode the presented information [3,4]. Mean effect sizes on fluency, set shifting, planning, and problem solving abilities point to large overall differences between OCD patients and healthy controls, indicating poorer performance in patients [3,4]. With regard to attention, some aspects such as attention span, sustained attention, and selective attention seem to be unaffected, whereas speed of information processing was impaired in about half of the studies. However, an analysis of the effect sizes for medicated and unmedicated samples has implicated, at least partially, the intake of psychotropic medications in slowing information processing in these patients [4].

It seems that core neural regions implicated in OCD pathophysiology might overlap with those involved in humor appreciation. In fact, exploration of the neuroanatomical mechanisms of humor appreciation has yielded evidence for the involvement of both the left and the right hemispheres. Specifically, one study reported activation of the right inferior frontal gyrus, the left superior temporal gyrus, the left middle temporal gyrus, and the left cerebellum [5]. Additionally, Ramachandran and Blakeslee [6] have proposed a 'laughter circuit' involving regions of the limbic system also involved in emotions (hypothalamus, mammillary bodies, and cingulate gyrus); moreover, the amygdala seems to play a key role in giving humor an emotional dimension [5]. Some evidence suggests that cognitive processing of humorous material involves different neural networks based on the modality (that is, verbal vs non-verbal), as well as the logical mechanisms (that is, visual puns, semantic or theory of mind cartoons) on which the stimuli are based. The right frontal region has been implicated in the appreciation of jokes, and both right and left frontal regions have been implicated in non-verbal cartoon appreciation [7]. Increased activation of the anterior medial prefrontal cortex, bilateral superior frontal gyri and temporal-parietal junctions has been reported during the processing of incongruity resolution humorous cartoons as compared with the processing of nonsense humorous cartoons [8]. Semantic humorous cartoons evoked the same pattern of brain activation as in incongruity resolution processing, while visual puns led to increased activation of extrastriate cortex and theory of mind non-verbal cartoons provoked increased activation of the medial prefrontal cortex and the temporal-parietal junctions [9].

Moreover, basic cognitive processes implicated in OCD pathophysiology might overlap with those involved in humor appreciation. The frontal lobes have been linked with several cognitive processes (that is, working memory, mental shifting, abstract thinking) that would appear to be critical in humor appreciation and expression. Researchers have found a significant relationship between performance on a verbal humor appreciation task and measures of working memory, mental shifting, and verbal abstraction, whereas a non-verbal humor appreciation task was related to working memory and visual search and scanning in patients with frontal lesions [7].

Given the neuroanatomical regions and cognitive functions involved in OCD and in humor appreciation, it is conceivable that patients with OCD might demonstrate impairment in this cognitive ability. Only one study to date, however, has explored humor appreciation in OCD [10]. In this study, unmedicated patients with OCD exhibited significantly slower initial velocities of involuntary laughing movements, as well as a lower frequency of laughing reactions, while watching a non-verbal humorous movie (*Mr. Bean*), than healthy controls. These findings could not be accounted for by differences in emotional reaction time and judgments of the funniness of the movie. Therefore, our purpose in undertaking the present study was to investigate the appreciation of incongruous details in the visual depiction of humor in a group of Greek patients with OCD.

## Methods

### Participants

Participants were 25 (ten male) outpatients with a diagnosis of OCD and 25 (14 male) healthy controls. Participants in the two groups gave their informed consent and experimenters adhered to international ethical standards relating to research participation. Patients with OCD were recruited from a community mental health center, while the healthy participants were recruited from the community by word of mouth. All patients were diagnosed according to *Diagnostic and Statistical Manual of Mental Disorders*, 4th edition (DSM-IV) criteria [11]. Diagnosis was confirmed with the Greek version (translation/adaptation to the Greek language by S Beratis) of the Mini International Neuropsychiatric Interview (4.4) (MINI) [12]. Severity of OCD symptoms was measured with the Yale-Brown Obsessive Compulsive Scale (Y-BOCS) [13,14]. At the time of the study, 18 patients were receiving antidepressants (six of them were also coadministered atypical antipsychotics), while the other seven were free of medication. Demographic characteristics of the two groups, as well as patient clinical data are presented in Table 1.

**Table 1:** Participant demographic characteristics, clinical data and test scores

Variables	Patients with OCD (n = 25)	Healthy controls (n = 25)
Age	32.68 (± 8.95)	33.40 (± 7.31)
Sex: M/F	10/15	14/11
Level of education (years)	12.44 (± 2.96)	11.96 (± 1.67)
Duration of illness (years)	14.08 (± 9.92)	-
Age of onset	18.28 (± 7.74)	-
Y-BOCS:		
Obsessions	11.56 (± 5.63)	-
Compulsions	9.44 (± 6.96)	-
Total	21.00 (± 11.35)	-
PHAT	16.92 (± 2.00)	16.32 (± 2.15)

OCD = obsessive-compulsive disorder; PHAT = Penn's Humor Appreciation Test; Y-BOCS = Yale-Brown Obsessive Compulsive Scale.

Exclusion criteria for both groups included neurological and developmental disorders, a history of head injury, alcohol or drug abuse during the 6-month period prior to testing, and any physical illness that may have affected their cognitive performance. Additional criteria for healthy participants were a history of a psychiatric disorder or treatment; all healthy participants were screened during a semistructured interview before entering the study.

### Humor appreciation

We administered Penn's Humor Appreciation Test (PHAT), a computerized test comprising 20 pairs of captionless cartoons by Mordillo [15]. His style of humor involved an incongruous detail in the visual depiction, specifically, an anomalous detail in an otherwise typical scene or the main character being unaware of a peculiar detail in his/her world to which the observer was privy. Consequently, appreciation of these cartoons required the perception of anomalous information and/or the comprehension of the depicted character's mental state. Each set of stimuli consisted of two almost identical drawings, one of which (the original) was funny, whereas the other was designed not to be funny by alteration of a central element in the cartoon. Each pair was presented as a single unit on a computer screen at the same time. For example, in one cartoon an astronaut has landed on an uninhabited planet and is putting up a flagpole to indicate that he is the first to land there, while in an identical cartoon, unbeknownst to him, he has landed on a big balloon. In another item, one cartoon shows two lovers kissing as their swings meet in the middle. In an identical cartoon, the two lovers' swings are completely covered by a spider web. Examinees made a forced choice for each pair, choosing which one of the two cartoons was funnier, or, alternatively, indicated whether they thought they were equally funny. The dependent measure was the number of correct responses defined as choosing the funnier of the two cartoons in the pair.

Normative data for the PHAT were based on 17 healthy normal volunteers and were used in the evaluation of the effects of a double-blind administration of three stimulant medications (caffeine, dextroamphetamine, and modafinil) or placebo on the ability to appreciate humor, including visual (cartoons) stimuli, following 49.5 h of sleep deprivation [16]. Moreover, the PHAT has been found to successfully discriminate patients with schizophrenia [17], but not remitted patients with bipolar disorder I [18], from healthy controls.

### Statistical analysis

Group comparisons (patients with OCD and healthy controls) were conducted with two-tailed t tests for continuous data, and with χ^2 ^tests for categorical data. A univariate general linear model was conducted in order to examine potential group and gender differences. We investigated correlations of performance on the PHAT with age of onset and duration of illness, as well as with the scores on the Y-BOCS by using two-way Pearson coefficients. Additionally, multiple stepwise regression analysis was performed using the PHAT score as an independent variable and the compulsion subscale and total scores on the Y-BOCS as dependent variables.

## Results

Patients with OCD and healthy individuals did not differ significantly in age (*t*(48) = 0.31, *P *= 0.76), level of education (*t*(48) = -0.71, *P *= 0.48), and male-to-female ratio (χ^2 ^(1) = 1.28, *P *= 0.26).

We found no effect for group (F(1,46) = 1.547, *P *= 0.22) or gender (F(1,46) = 2.038, *P *= 0.16), or a group × gender interaction (F(1,46) = 0.038, *P *= 0.84) on PHAT performance (Table 1). Patients with OCD receiving treatment with selective serotonin reuptake inhibitors (SSRIs) did not differ significantly from patients who were medication free (t(23) = -1.254, *P *= 0.222).

Correlations within the OCD group failed to reveal any significant relationships between PHAT performance and age of onset (*r*(25) = -0.17, *P *= 0.41) or duration of illness (*r*(25) = -0.17, *P *= 0.41). Regarding severity of psychopathology, as measured by the Y-BOCS, performance on the PHAT yielded a significant negative correlation with the compulsion subscale (*r*(25) = -0.44, *P *= 0.027) and total score (*r*(25) = -0.40, *P *= 0.048), but not with the obsession subscale (*r*(25) = -0.26, *P *= 0.21). The compulsion subscale (B = -0.127) had a significant influence on PHAT performance (ΔR = 5.548, df = 23, *P *= 0.027), explaining 19.4% of the total variance.

## Discussion

We found that humor recognition in captionless cartoons by a group of patients with OCD did not differ from that of age-matched, education-matched and gender-matched healthy controls. To the best of our knowledge, this study is only the second addressing humor appreciation, an exclusively human ability that contributes to building and maintaining social relationships [19], in OCD, a disorder with detrimental effects on social relationships and role functioning [20]. Similarly, Mergl *et al*. [10] found no differences between OCD patients and healthy individuals in their assessment of the funniness of non-verbal humorous movies, a variable that is similar to the procedure involved in the PHAT. Unmedicated patients with OCD in their study, however, were slower in showing initial involuntary laughing movements and had fewer laughing reactions than the control group, which were normalized after 10 weeks of treatment with sertraline [10]. In our study, no differences were observed on the PHAT score between patients receiving and those not receiving SSRI medication.

In the present study, performance on humor appreciation correlated with severity of compulsion among patients, with poorer performance associated with increased severity. Our findings were partially in accordance with those of the Mergl *et al*. study [10], where total and compulsion Y-BOCS scores were found to correlate with the assessment of the 'funniness' of *Mr. Bean *movies. They also reported less frequent laughing as OCD symptom severity increased.

Both genders share an extensive humor response strategy as indicated by recruitment of similar brain regions in men and women: the temporal-occipital junction and temporal pole, as well as the inferior frontal gyrus. However, women activate the left prefrontal cortex and mesolimbic regions, including the nucleus accumbens to a greater degree than men. These results indicate sex-specific differences in the neural response to humor [21]. However, gender had no effect, nor did it interact with group, on PHAT.

Humor processing probably involves distinct networks for the cognitive and affective component of humor [22]. It has been proposed that the dorsolateral prefrontal cortex might mediate comprehension of the cognitive component of humor recognition, while the medial prefrontal cortex might mediate the affective component of humor appreciation [23]. Moreover, in a recent functional imaging study, the medial prefrontal cortex was suggested as part of a network involved in processing the affective component of humor [24]. Available evidence from neuroimaging in patients with OCD has pointed to an orbitofrontal dysfunction rather than a dorsolateral or medial ventral prefrontal cortical involvement [1,2]. Also, neuropsychological studies [1,4] have suggested that performance of patients with OCD is impaired compared to those of healthy controls on set shifting tasks that are believed to be primarily sensitive to orbitofrontal dysfunction (Object Alternation Test [25] and Delayed Alternation Test [26]) and not to dorsolateral prefrontal damage (Wisconsin Card Sorting Task [27]). The PHAT is a task in which examinees are instructed to choose which one of the two cartoons that are presented to them is the funnier, thus, presumably, it is related to the affective humor appreciation component and, consequently, to medial ventral prefrontal cortex function. As dysfunction of the medial ventral prefrontal cortex is not ubiquitous in OCD, it was not surprising that patients with OCD responded to the PHAT in a manner similar to that of healthy controls.

The present study has certain limitations. Given that the MINI does not give a detailed evaluation of all DSM-IV axis I and II diagnoses, a possible comorbidity of OCD with other mental disorders, specifically anxiety or personality disorders, cannot be ruled out. Moreover, the evaluation of healthy controls was not based on a structured interview, leaving the possibility that some past or present mental disorders were not detected. Finally, no information about the general intellectual or specific cognitive functioning that may influence humor understanding regarding our research participant was available.

In summary, while humor appreciation based on captionless cartoons is not deficient in OCD, it appears to be related to illness characteristics.

## Competing interests

The authors declare that they have no competing interests.

## Authors' contributions

VPB designed the study, participated in data analysis and in writing the paper. MHK and MG participated in data collection and management. AA and KF participated in conceiving and designing the study and interpreting the data. XG participated in interpreting the data and writing the paper. GG participated in data analysis and writing the paper.
